# Time Burden of Electronic Medical Records on Nurses and Physicians in Saudi Arabia: Occurrence, Predictors, and Challenges—A Mixed-Methods Study

**DOI:** 10.3390/healthcare14040441

**Published:** 2026-02-09

**Authors:** Ali Mohammed Al-Yasin, Homood A. Alharbi

**Affiliations:** College of Nursing, King Saud University, Riyadh 12372, Saudi Arabia; homalharbi@ksu.edu.sa

**Keywords:** electronic medical record, health personnel, workload, nursing staff, Saudi Arabia, time factors, documentation, medical informatics

## Abstract

**Background:** Electronic Medical Records improve decision-making but add administrative burdens for healthcare providers, such as physicians and nurses. While the rate of adoption is high in Saudi Arabia, the concrete temporary impact and reasoning behind their adoption are understudied. **Objectives:** This study is a Mixed-Methods Study designed to ascertain the number of hours of EMR use among physicians and nurses, the predictors of using EMRs for extended periods, perceived barriers and clinical impacts. **Methods:** A sequential mixed-methods study was performed in three hospitals in Riyadh, Dammam, and Makkah. Quantitative data from 503 clinicians were analyzed using inferential statistics, followed by thematic analysis of 10 semi-structured interviews. **Results:** A total of 503 professionals (162 physicians, 341 nurses) participated. The majority were females (67.2%), aged 30 to 40 years (44.9%), and non-Saudi (62%). Nurses reported a significantly higher daily EMR workload than physicians with 5.43 h (45.25%) versus 4.34 h (36.17%), with a mean difference of 1.09 h (t = −5.76, *p* = 0.001). Ordinal logistic regression identified female gender, non-Saudi nationality, nursing position, and lack of advanced education (Masters/Doctorate) as high-significance predictors of prolonged usage (all *p* < 0.005). Additionally, years of experience (*p* = 0.001) and EMR training (*p* = 0.003) were significant factors. Perceived barriers were moderate but significantly predicted by professional position (*p* = 0.004), work region (*p* = 0.017), and training duration (*p* = 0.001). Qualitatively, thematic analysis revealed four major barrier categories: system performance, infrastructure issues, lack of IT support, and increased workflow burdens. While EMRs improved professional practice and patient safety by solving handwriting issues and structuring data, they forced work routine adjustments that significantly reduced bedside patient interaction and assessment time.

## 1. Introduction

In the modern healthcare landscape, Electronic Medical Records (EMRs) have revolutionized clinical practice by replacing conventional paper systems with a digital technology infrastructure, designed for increased accuracy, accessibility and efficiency [1,2,3]. By giving immediate access to longitudinal patient data, these systems help with clinical decision-making based on evidence, as well as patient care based on quality [4,5]. In the Kingdom of Saudi Arabia, this digital transition has been accelerated by the national, electronic health-led programs of the Ministry of Health. These strategic programs are aimed at creating a unified digital framework within the public and private sectors by converting the nationwide healthcare architecture into an electronic format [6,7].

Despite these systemic advancements, introducing paper systems into the digital world has had significant unintended consequences that prevent medical systems from realizing the benefits of EMRs. A major concern is the heavy time load for primary users, namely physicians and nurses [8,9]. The global literature has consistently indicated that healthcare professionals currently spend a disproportionately large amount of time managing electronic documentation relative to direct patient care. This issue not only impacts the clinical workflows but also acts as a catalyst for professional burnout, leading to emotional exhaustion, increased stress, and reduced job satisfaction [10].

Regarding the rapid digital expansion in Saudi Arabia, these issues are compounded by certain unique sociocultural and structural factors. While global research has thoroughly described the burden of EMRs in comparatively homogenous settings in the Western world, there is a critical knowledge gap regarding the manifestation of these pressures in highly multinational workforces such as that of Saudi Arabia. There is preliminary evidence that linguistic diversity and regional infrastructure may be unique characteristics that exacerbate medical professionals’ digital workload [11]. Furthermore, despite the discussion on EMR usability, the precise predictors of the time burden and the training paradox in which increased instruction hours can paradoxically increase perceived documentation fatigue remain significantly under-explored in the region [12]. This fragmentation of the current knowledge acts as a barrier to creating strategies that are evidence-based and specific to the Middle Eastern healthcare environment.

**Figure 1:**
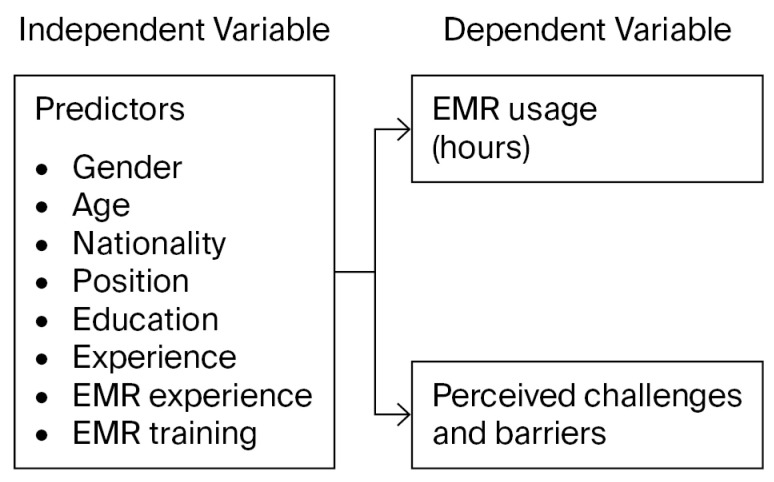
A conceptual framework showing the relationship between independent predictors and EMR usage outcomes. Adapted from [13].

Consequently, this study aims to fill in these gaps by quantifying the EMR time burden and discussing the underlying factors that result in system-related fatigue for physicians and nurses in Saudi Arabia. This research is guided by a conceptual framework based on the Technology Acceptance Model, which suggests that technology-related behavior is influenced by user perceptions such as useful and ease of use [13]. As identified in Figure 1, this study assumes that EMR usage time and perceived barriers are determined by a combination of independent variables, such as person properness, professional position and technical preparation through EMR training and experience. Using a mixed-methods approach, this study attempts to quantify the average monthly hours of extended use of EMRs, identify demographic predictors of extended digital system use, and qualitatively understand barriers to ensuring the quality of healthcare services. Through this investigation, this research will contribute vital information for informed decisions to guide national health policies, promote digital health sustainability, and improve the wellbeing of frontline healthcare providers.

## 2. Materials and Methods

### 2.1. Study Design, Setting and Duration

This study employed a sequential mixed-methods design to address the research objectives. The quantitative part employed a correlational cross-sectional design and was used to evaluate patterns of EMR use, compare the application of EMRs between nurses and physicians, and identify significant demographic predictors. This was followed by a qualitative phase used to explore the perceived barriers and challenges related to EMR use and the impact of EMRs. This study was conducted across three major regional hospitals under the Security Forces Hospital Program in Riyadh, Dammam, and Makkah from January to October 2025.

### 2.2. Study Population and Eligibility Criteria

The study population comprised all nurses and physicians from the Security Forces Hospitals in Riyadh, Dammam, and Makkah. The inclusion criteria required participants to have at least three months of work experience to ensure sufficient familiarity with the EMR system, and only those nurses and physicians who directly utilized the EMR system as part of their daily professional responsibilities were considered eligible. Those with less than three months of clinical experience, as well as administrative and non-clinical staff, were excluded to maintain focus on those directly engaged in patient care and documentation.

### 2.3. Sampling Technique

For the quantitative study, a multistage sampling technique was used to select a representative sample of physicians and nurses working in Security Forces Hospitals. The study population was stratified into six categories, namely professional role, hospital, regional affiliation, nationality, clinical environment, and seniority level. This stratification ensured diverse representation across professional, institutional, cultural, and clinical dimensions. By including these distinct categories, the study design safeguarded against the over-representation or under-representation of specific groups, thereby strengthening the external validity and generalizability of the findings.

For the qualitative study, a purposive sampling technique was employed to select ten participants from the three hospitals [14].

### 2.4. Sample Size Calculation

The sample size was calculated using an online tool called Epitools, which helps to compare two proportions [15]. The main inputs to the calculation were obtained from the literature, which states that physicians spend approximately 21% of their working time on EMRs, while nurses spend about 35% [16]. These percentages were used to define the expected absolute difference of 14% between the two cohorts. The computation was based on a two-sided significance of 0.05, a statistical power of 0.80, and a ratio of 1.3:1 between nurses and physicians, which is the approximate ratio of nurses to physicians in the clinical facilities. With these parameters, the sample sizes had to be at least 156 physicians and 203 nurses, resulting in 359 participants. By adding a 20% non-response rate, the sample size was increased to 195 physicians and 254 nurses.

### 2.5. Data Collection Tool

#### 2.5.1. Quantitative Tool

The quantitative data were collected using a structured questionnaire adapted from four previously validated studies [17,18,19,20] and refined to align with this study’s objectives. The questionnaire consisted of 30 questions divided into five sections (Supplementary File S1). The first section collected demographic information and comprised 11 items, with some items tailored exclusively for either nurses or physicians. The second section (EMR usage) consisted of two items. The first item measured the average number of hours per shift/day spent using the EMR system, with response options ranging from “Less than 1 h” to “More than 6 h.” The second item explored the range of clinical tasks performed through the EMR system, allowing respondents to select multiple applicable functions. The third section (EMR training and support) comprised three items used to assess participants’ EMR training experience, total training hours, and perceived adequacy and challenges encountered while using EMRs. The fourth section (perception of EMR use) consisted of eleven items addressing the barriers and challenges related to EMR systems, as well as their perceived impacts on patient care and job satisfaction. The final section (suggestions and recommendations) comprised three open-ended items designed to elicit qualitative insights from participants regarding their experiences with EMRs.

The draft questionnaire was evaluated for content validity by five experts working in the fields of nursing, epidemiology, health systems management, and EMR specialisms. Each item was rated as being essential, useful, or not essential. Using Lawshe’s method, most of the items (85%) achieved the minimum Content Validity Ratio. Extensive expert consensus and significant revisions through feedback confirmed the content validity of the questionnaire and the final instrument was thorough, relevant and in line with the aims of this study. The questionnaire then underwent a face-validity assessment with a focus group of the five nurses and five physicians examined to test the clarity, readability and usability of the items. Feedback on ambiguous and problematic items was systematically analyzed, leading to improved clarity and reduced respondent burden, and suitable to the clinical situation. The internal consistency of the EMR Time Burden Questionnaire was assessed for 11 structured items (Questions 17–27) using Cronbach’s alpha, giving an overall standardized α of 0.78 (95% CI: 0.64–0.88), indicating satisfactory internal reliability.

#### 2.5.2. Qualitative Tool

Qualitative data were collected using a semi-structured interview guide consisting of three questions, namely the hours spent using EMR per shift, challenges and barriers experienced while using EMRs, and the impacts of EMR usage on the respondent’s patient care quality. The researcher asked follow-up questions or additional questions based on the respondent’s feedback, especially if the respondent’s feedback was insufficient.

### 2.6. Data Collection Procedure

For the quantitative phase, the structured questionnaire was hosted online on Google Forms. Upon receiving ethical clearance from the hospital IRB committees, the researcher coordinated with them for identifying the eligible population and stratifying participants proportionally as nurses and physicians. Within each stratum, participants were selected using spreadsheet-based randomization. Next, the IRB facilitated the secure distribution of the questionnaire link exclusively to the randomly selected staff members. The questionnaire remained open until the target sample size of 359 participants was achieved (comprising 156 physicians and 203 nurses).

For the qualitative phase, data were collected by conducting short interviews with five nurses and five physicians at the Security Forces Hospitals who had at least ten years of experience in this environment and had used the EMR system for at least five years within their respective fields. Additionally, the selected participants held senior positions within the hospital; these participants were selected to ensure that they carried greater responsibility and accountability when using the EMRs. Participants were invited via formal emails, which included the researcher’s identity, study title, and objectives. Interviews were conducted at the respondent’s hospital and recorded using a phone voice recorder. The interview began with the researcher providing an overview of this study and confirming the participant’s willingness to participate, and only proceeded after receiving the respondent’s approval.

Qualitative data collection was concluded after interviewing ten participants because data saturation had been achieved. At this stage of the research, the interviews yielded consistent patterns regarding EMR challenges and professional burdens, indicating that further data collection would not produce new thematic insights.

### 2.7. Data Analysis

#### 2.7.1. Quantitative Data Analysis

Quantitative analysis was performed to describe patterns of EMR use and significant predictors of engagement. Continuous variables are reported as mean ± standard deviation (SD), and categorical variables are reported as percentages. Comparative analysis was conducted using a *t* test for continuous data. To assess demographic factors related to prolonged EMR use, ordinal logistic regression analysis was conducted. Additionally, multiple linear regression was used to examine contributors to perceived challenges and barriers. Statistical significance was defined as the *p* value being less than 0.05 and instrument reliability was checked using Cronbach’s alpha.

#### 2.7.2. Qualitative Data Analysis

Qualitative data were analyzed using NVivo 15 software to conduct a thematic analysis, chosen for its ability to efficiently manage, organize, and code large volumes of qualitative data. Thematic analysis followed the six-phase process outlined by Neuendorf et al. (2018) [21]: 1. Familiarization—repeated reading of transcripts for immersive understanding. 2. Initial Coding—assigning short labels to relevant data excerpts. 3. Theme Development—grouping related codes into potential themes. 4. Theme Review—refining themes to ensure internal consistency and external distinction. 5. Theme Definition—clearly naming and defining each theme. 6. Reporting—presenting findings in relation to the research questions, supported by direct quotes [21]. Codes were assigned based on the three questions asked in the interview. The first question concerned for how long the participant had used the EMR system. The second question concerned the challenges and barriers faced when using the EMRs. The third question assessed the impact of using EMRs. Two codes were identified that were related to EMR barriers and challenges based on a past literature review, namely delays and their time-consuming nature. Besides these two codes, inductive thematic analysis was carried out to identify new codes for each of the questions that aligned with this study’s research objective.

To make sure that the findings were accurate and reliable, this study followed four key steps to establish trustworthiness. First, the researcher conducted member checking by presenting the notes made from the interview to the participants, thereby ensuring the we correct recorded their views. Second, detailed records of every step taken during the research were kept as an audit trail to prove that the final results came directly from the words of the nurses and physicians rather than personal opinions. Third, the research was thorough in its descriptions of the particular settings of the two hospitals and the various backgrounds of the staff involved, helping others to see how these findings may apply differently to various clinical settings. Finally, the research team collaborated to examine the coding process and come to an agreement on the final themes, ensuring that the analysis was balanced and objective.

Regarding the qualitative sample size, data saturation was tracked during the interview process to ensure the depth and richness of the findings. By the eighth and ninth interviews, the research team noticed a high level of repetition in the identified challenges and barriers and that no significant new themes emerged. The final sample of ten participants was, therefore, considered sufficient to reach thematic saturation in this particular professional context. This approach is consistent with the accepted guidelines for qualitative research, which maintain that for a narrowly defined study population with focus on research, a sample size of between eight and twelve participants is often sufficient to achieve saturation.

### 2.8. Conceptual Framework

The theoretical framework used in this study is based on the assumption that the time clinicians spend on EMRs is influenced by a combination of personal characteristics, technical exposure and preparation, and environmental and contextual obstacles. Although this framework is informed by the Technology Acceptance Model (TAM), which explains technology-related behavior through perceived usefulness and perceived ease of use [22], this study does not aim to test TAM or directly measure its core perceptual constructs. Instead, TAM is used as a conceptual reference to support the general premise that user characteristics and contextual factors shape technology-related behavior. Accordingly, this study adopts a usage-oriented perspective, focusing on observable EMR usage (time spent) and perceived challenges and barriers rather than on users’ attitudinal or intention-based perceptions. The framework therefore guides the formulation of the research objectives and provides a basis for interpreting both quantitative and qualitative findings (Figure 1).

### 2.9. Ethical Consideration

Ethical approval for this study was obtained first from the King Saud University Institutional Review Board (KSU-HE-25-457) and then from the Security Forces Hospital IRB board (25-786-06). All data were only used for academic purposes. For the questionnaire phase, informed consent was sought, participation in this study was voluntary, and the purpose of this study was explained to the participants prior to consent. No identifying information was requested in the questionnaire (e.g., names or ID numbers) to maintain anonymity. For the interview phase, participants were approached individually and briefed about the aims and scope of the interviews and the voluntary nature of their participation. Prior to the start of the interviews, verbal consent was taken. Interview recordings were securely stored and erased after transcription and analysis.

## 3. Results

### 3.1. Quantitative Data Results

A total of 503 professionals (including 162 doctors and 341 nurses) participated in this study. Table 1 indicates that most of the respondents were female (67.2%). Physicians were predominantly males (57.4%), while the majority of nurses were females (78.9%). The majority of the participants were aged between 30 and 40 years (44.9%). Regarding nationality, 62% of respondents were non-Saudi nationals. Regarding the positions of the physicians, a plurality were residents (42.6%). Regarding the primary specialties of the physicians, a plurality specialized in pediatrics (27.2%). Regarding nurses, a majority were front-line. Regarding educational level, most respondents (65%) had a bachelor’s degree. Regarding years of experience in healthcare, a plurality of the participants (36%) had 10 to 20 years of experience in healthcare. An almost similar percentage (39.8%) reported having 1 to 5 years’ experience of using EMRs. Lastly, the regional representation of participants indicates that 50.1% were located in Riyadh, 26.2% in Makkah, and 23.7% in Dammam, which is a balanced geographical representation of the three hospital locations.

Table 2 illustrates a distinct divergence in EMR usage patterns across professional roles. Nurses were the most frequent users, with 53.7% spending more than 6 h daily on the system. In contrast, physician usage was concentrated primarily between 3 and 4 h per shift. While 87.1% of staff received formal training, the duration was limited, with 48.9% receiving less than 5 h. Reported obstacles also varied by role: physicians primarily cited the administrative burden of data entry, whereas nurses identified technical infrastructure issues, such as system latency, as their primary barrier.

Table 3 presents the distribution of EMR usage hours by professional role. The data show that the majority of nursing staff fall into the highest usage bracket, with more than 6 h per day. In contrast, physician usage is primarily distributed across the middle brackets (3–4 h). When calculating total daily use via midpoint estimation, nurses recorded the highest cumulative time spent on the electronic system across all other healthcare categories.

Table 4 shows that the finding that nurses spend 41.73% of their total professional time on EMR activities is significantly higher than that of their physician counterparts. This indicates an unequal distribution of the administrative workload. This disproportionate digital footprint suggests that the current technical infrastructure may be in direct conflict with other bedside clinical priorities. Furthermore, the high standard deviations observed (SD ≈ 2) indicate that, although the group average is high, the digital burden is not experienced uniformly. This variability implies that certain clinicians may be facing even more extreme documentation demands, potentially leading to role-induced fatigue and a shift in professional identity from care provider to data manager.

The regression analysis in Table 5 shows that the digital workload is not a neutral outcome of technology adoption but is deeply stratified by demographic and institutional factors. The high odds ratios for females and nurses (OR ≈ 3.0) suggest a gendered and role-based clerical tax, with documentation duties falling disproportionately on these cohorts. This may reflect the nature of nursing documentation protocols or cultural factors within the clinical hierarchy. Furthermore, the finding that advanced education (Master’s and PhD) significantly reduces the odds of prolonged usage (OR < 0.5) is particularly salient. This suggests that advanced training may facilitate more efficient data synthesis or that those in higher academic positions have more autonomy to delegate documentation tasks. Finally, the peak in usage among mid-to-late-career professionals (10–20 years) suggests a digital gap in which these clinicians may have high clinical responsibility but lack the digital-native fluency of younger staff, leading to longer interaction times to complete the same tasks.

Table 6 summarizes the perceived barriers across professional roles. Analysis of institutional support indicates that nursing staff reported higher levels of training and technical assistance than physicians. Conversely, physicians reported a stronger belief that digital systems improve the quality of care, with a mean score of 3.91 compared to 3.60 for nurses. While experience in a healthcare setting yielded significant results regarding digital efficacy, no significant difference was observed between groups regarding job satisfaction related to EMR use.

Table 7 shows that the multiple linear regression analysis confirms that the challenges associated with digital systems are mainly driven by institutional and professional factors rather than individual demographics. The overall model is statistically significant and explains 11.8% of the overall variance in perceived barriers. Among the predictors, professional position and hours of training have the most influence on the results. The positive correlation with training implies that staff receiving more training during formal instruction may give them more acute awareness of parts of the system or allow them to be assigned to additional documentation intensive roles. Additionally, work region shows a significant negative relationship, with the specific institutional setting and local infrastructure being critical in determining the level of difficulty encountered by staff. Factors such as gender, age, education level, and general healthcare experience did not contribute significantly to the model. These results suggest that strategies aimed at minimizing clinical barriers should be based on the optimization of job-specific workflows and regional technical support, rather than on personal characteristics.

### 3.2. Qualitative Data Results

#### 3.2.1. Demographic Profile

The ten respondents assessed in this study were employed at Security Forces Hospitals located in Riyadh, Dammam, and Makkah. Five of the participants were nurses, holding positions such as registered nurse and head nurse. The remaining five respondents included registrars, consultants, and senior staff midwives. The participants’ ages ranged from 32 to 57 years, working experience ranged from 10 to 35 years, and education level ranged from diplomas to PhD.

#### 3.2.2. Hours of EMR Use

Data analysis revealed that EMR use has become an ordinary aspect of clinical work that takes up between 3 and 7 h of an 8–12 h shift. Respondents highlighted that this online work now influences much of their professional activity. Participant 7 (P7) described the situation, saying that during their 12 h shift, half a day (between six and seven hours) was devoted to completing this documentation. P3 and P9 reported similar time demands, revealing that over half of clinician shifts are occupied by screens rather than patients. This degree of engagement represents role alteration, with administrative data entry tasks competing directly with bedside care.

Moreover, the shift to digital systems does not seem to have simplified documentation; rather, it seems to have increased its use. This issue was especially pronounced when current needs regarding digital requirements were compared with past needs regarding manual records. Participant 8 stated that she now spent five hours on EMRs, whereas she previously spent significantly less time writing by hand. This implies the EMR is a fixed workspace that dictates the pace of clinical work and increases the cognitive load.

New patient documentation was identified as consuming about half of the appointment time per patient. Participant 6 clarified as follows: “when the interview time is small such as 10 to 20 min, the documentation will occupy 50 percent of the time. Documentation always took close to 20 min even during a 45 min appointment.” This ratio indicates that technical requirements and checklist completion, which P7 claimed required about 15 min per patient, are now as challenging as the clinical assessment itself. These trends show that there has been an imbalance in structures, with administrative practice taking up more time than direct provider–patient communication.

#### 3.2.3. Challenges and Barriers Related to EMRs

**Figure 2:**
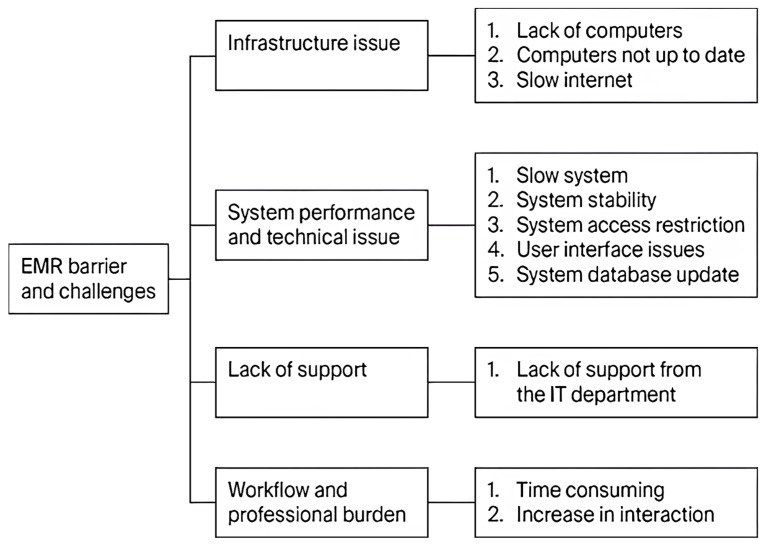
A thematic framework for identified barriers and challenges related to EMR implementation.

Regarding EMR challenges and barriers, twelve codes were identified, which were grouped into four themes, namely infrastructure issues, system performance and technical issues, lack of support, and workflow and professional burden. Figure 2 and Table 8 provide an overview of the codes and themes identified for barriers and challenges related to EMR.

##### Infrastructure Issues

Physical infrastructure is crucial to the implementation of EMR systems, but the participants documented significant disparities in hardware accessibility and functionality. One of the major obstacles was the unavailability of adequate computers, as the staff competed for access to the few available workstations. According to Participant 9 (P9), the unit has a limited number of computers. The lack of workstations transforms the EMR into a support tool, and it becomes a structural barrier due to inevitable delays in real-time documentation.

Moreover, clinical work was further slowed because of the age and quality of the available devices. The participants claimed that even the EMR user experience involved certain problems with hardware since the computers were outdated (P2). The old hardware is indicative of the fact that infrastructure has not kept up with digital requirements and employees are left to operate in slow and unreliable systems.

Poor connectivity has also weakened system reliability. Issues with internet connectivity and poor connectivity were commonly reported by various participants such as P1 and P8, who reported that they experienced internet problems frequently and sluggish workflows. Such technical flaws mean that clinicians cannot count on the stability of the systems. Consequently, a lack of workstations, outdated technology, and poor connectivity form the weak infrastructure that makes systems less reliable and negatively affects the productivity of staff.

Thus, these problems reflect discrepancy between digital demands and concrete ability. A lack of adequate infrastructure acts as a hindrance to EMR use. Thus, inefficient investment in hardware and networks negatively affects efficiency and jeopardizes the sustainability of digital transformation.

##### System Performance and Technical Issues

Delays in the system and regular downtime were also a threat to clinical continuity. One subject mentioned having a slow system and a delay in the command response times (Participant 1), and the rest had experienced crashes because of irregular updates. Participant 2 stated that the system was effective approximately 95 percent of the time, i.e., any minor failure created secondary work and stress. This demonstrates that the performance needs of high-risk medical settings, where system instability may compromise trust in digital tools, have not been adequately addressed by technical management.

There were additional administrative burdens caused by restrictive system rules and unfinished interface design. The existing protocols tend to restrict some data entries to consultants alone, as Participant 2 noticed: “certain entries are only meant to be entered by consultants only.” Such limitations limit cooperation and prevent positive decision-making. Moreover, the poor human-centered design manifests itself in repetitive navigation between various screens, i.e., “moving between one icon to another” (Participant 5). Rather than aiding clinical judgment, the interface transforms tasks into fill-out drills.

These results suggest that technical instability and inflexible system architecture not only slow down work but also transform the clinical role into an administrative role. The EMR system forces the clinician to overcome the flaws of the system on the spot by focusing less on users and more on technical operations. Such rigidity restricts the capability of the system to accommodate healthcare work and creates an overburdened workforce instead of an empowered one.

##### Lack of Support

Poor technical support was strongly related to operational challenges in the EMR environment. Respondents said that IT teams responded slowly, which slowed down their work. Participant 4 explained that this team was very slow in responding to correct issues in the system. Participants 9 and 10 observed that long waits were frustrating and interfered with the flow of work.

Many system updates are handled by an external party and leave gaps that clinical staff cannot address independently. Participant 1 indicated that updates have been provided that they cannot understand due to a lack of IT department support. These delays to work activities can transform minor technical issues into significant obstacles. On the whole, this inability to provide timely support and maintain the system does not allow staff to work efficiently in a high-stakes environment and forces them to deal with technical problems while treating patients.

##### Workflow and Professional Burden

The digital transition has altered workflow patterns, as EMR systems may be more time-consuming compared to paper records. Participant 5 explained that the EMR takes longer to complete tasks than the time previously taken to complete paperwork because it requires strict documentation and has strict requirements in terms of the time taken to complete notes and orders. This demonstrates how system design might focus on administrative functions at the cost of clinical workflow, leading to data duplication and fragmented, piecemeal work. Participant 6 also mentioned that EMRs are time-consuming since, without shortcuts or dictation, the staff have to manually type each field. This adds to the perception that EMRs are more time-consuming than past systems.

In addition to time wastage, EMR use has burdened the profession by necessitating interdepartmental communication to address documentation problems. Participant 4 described how some departments became overworked because of the many calls between the nurses and doctors regarding documentation issues. The EMR system has introduced new bureaucracies instead of making communication easier. Overall, these issues imply that EMR systems have made clinical work more administrative, leaving less time to work with patients directly and adding more unnecessary complexity to day-to-day working processes.

#### 3.2.4. Impact of EMR Usage

**Figure 3:**
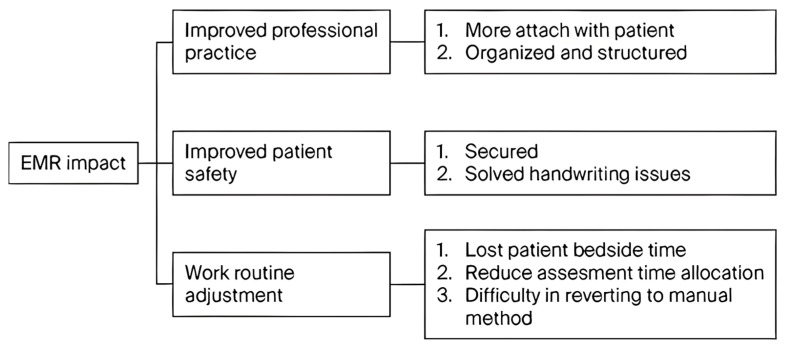
A thematic analysis of the perceived impact of Electronic Medical Records.

As shown in Figure 3 and Table 9, seven codes were regarding the impact of using EMRs, which were grouped into three themes, namely improved professional practice, improved patient safety, and work routine adjustment.

##### Improved Professional Practice

The implementation of EMR systems enhanced the structure of clinical documentation, making it more organized and adaptable to international standards, strengthening the local workflow. Participant 2 observed that documentation, when completed online, was more systemized and organized and met international standards, unlike manual systems that tended to be impacted by illegible handwriting and a lack of readability. The system has facilitated this because it can maintain collected data in distinct chronological order (Participant 3), whereby patient histories can be documented in a rational and reachable order. The EMR helps to facilitate improved and more reliable and consistent reporting on the part of professionals because ambiguity is replaced with standardized documentation.

Clinical operations have been enhanced beyond documentation clarity because of the centralization of information. Participant 4 reflected that due to the availability of all information in a single system, it is easier to manage patients, as the EMR provides a full picture of the care provided to patients. The provider–patient relationship also seems to be influenced by the availability of full data. Participant 1 shared that the availability of more detailed records enabled them to feel more attached to and familiar with the patient. This implies that having available data not only facilitates administrative work but also assists clinicians in building a better picture of patient histories. Overall, these enhancements suggest that an intertwined digital system is fundamental for ensuring clinical quality and professional continuity.

##### Improved Patient Safety

EMR systems have also enhanced patient safety. According to the respondents, digital documentation improves the protection of patient information, enhances transparency and accountability, and maintains legal safeguards regarding documentation and access. Participant 7 explained that the structured records are useful in formalizing clinical practice, which implies that EMR offers a legal shield for formalizing procedures while safeguarding confidentiality.

The shift to using electronic records has also solved the issue of poor handwriting that affected paper systems. Participant 6 described how, when made electronically, it is much easier to read notes, which enhances the comprehension of clinicians regarding the patient’s previous experience and the continuity of care. Similarly, Participant 10 noted that due to the clarity of doctor orders, the chances of making medication errors are minimal. EMR systems ensure that potential human interpretation errors are eliminated via reduced ambiguity and enhanced clarity in communication, giving way to standardized documentation and accountability within a system. Collectively, these results demonstrate that EMR systems have the potential to improve patient safety due to improved documentation, traceability, and communication among clinical teams.

##### Work Routine Adjustment

EMR systems have also led to alterations in routine work. Participant 5 described how much of the transition focused on patient safety with regard to data protection, data access control, and documentation practices aimed at facilitating legal compliance. Over time, these characteristics have affected the way in which clinical work has been organized and staff have changed their routines to reflect the digital processes.

According to Participant 7, there are also more intensive documentation requirements that enhance legal and organizational protections. EMR systems digitize the recording of actions and decisions, predisposing more accountable professional behavior. These adaptations, although at times challenging, signify that digital systems are proactively redefining the way in which clinicians plan, document, and authenticate their efforts.

## 4. Discussion

This paper explored the sociotechnical aspects of EMR usage by healthcare practitioners and revealed that digital documentation is no longer a secondary activity but a primary clinical activity. The analysis showed that there was an evident role-based difference in the use of EMR systems: nurses devoted 41.73 percent of their working hours to the use of EMR systems, whereas physicians devoted 33.38 percent. This difference was statistically significant (*p* = 0.001). Limited training and support also increased the time burden, as nearly half of the participants reported receiving less than five hours of formal training. Documentation pressure, a lack of support, and technical obstacles demonstrate that EMR systems have the potential to support and restrict practice at the same time. Although they enhance legibility, standardization, and data protection, they can also decrease the time available for bedside care.

The central value of this study is that it presents an integrated sociotechnical evaluation of EMR systems. In contrast to the studies primarily concerned with statistics for EMR usage, this study examines the influence of demographic factors on EMR engagement in a Saudi Arabian healthcare setting. The conclusions describe how digital transformation has transformed professional roles. In this case, the nurses involved in this study experienced significant workload changes, and the use of EMR constituted up to 41 percent of their working days. On a larger scale, this study offers a paradigm through which unstable systems, inflexible software design, and administrative pressures may restructure clinical activity into bureaucratic action in varied healthcare environments.

### 4.1. Mean EMR Use Hours for Physicians and Nurses

In this study, physicians spent an average of 4.34 h per shift using EMRs, while nurses spent 5.43 h, suggesting a relatively higher EMR workload for nursing staff. Compared with international studies, the results of this study are firm and contradict previous findings, demonstrating the diversity of EMR use patterns across healthcare systems. For instance, lower daily EMR use was reported in studies from the United States [23,24,25,26,27], Japan [28], and Jordan [29]. The difference can be attributed to the tertiary-care setting of this study, the complex and multidisciplinary care provided to patients, the broader range of EMR functions, the inclusion of nurses and physicians across multiple clinical departments, and higher patient volumes.

However, some studies have reported similar or even higher levels of EMR engagement. For instance, Goldstein et al. (2018) reported an average of 4.3 h per day of EMR use [30]. The comparably high duration may be due to the comprehensive EMR system supporting a wide range of clinical and administrative tasks. Moreover, the research was conducted at Oregon Health and Science University, a large tertiary hospital with high patient volumes and complex care delivery units. This institutional resemblance to Security Forces Hospitals suggests that EMR engagement levels may be similar, given similar workflow requirements and the complexity of patient care. The similarity between the two studies validates the present findings and shows that frequent EMR use is not specific to the Saudi setting but rather a global trend in technology-based healthcare settings. Another study reported that residents spent 5.38 h/shift using EMR systems, although it considered shifts of 12 h and units where patient acuity levels were higher [31].

The apparent difference in the number of hours spent suggests that digital infrastructures have begun to shift nurses’ responsibilities toward data management. While physicians use the system for episodic medical decision support, nurses spend 41.73% of their working hours in the system, suggesting a shift away from direct patient supervision toward more administrative patient care responsibilities. This suggests that the system, rather than facilitating care, may act as a bottleneck. When accessing documentation in the system takes more than 5 h out of a 12 h shift, the ability to perform clinical interventions at the bedside is limited.

The fact that this digital burden continues to exist globally suggests that current versions of digital systems cannot accommodate nursing workflows that contain a higher-than-average volume of documentation. The data point to an important phenomenon whereby the seemingly unrelenting technical requirements of digital systems overshadow their clinical purposes, thereby leading to system-induced burnout and the erosion of the provider–patient relationship.

### 4.2. Differences Between EMR Use Among Physicians and Nurses

The most salient observation of this study is the significantly higher EMR use recorded among nurses compared to physicians (*p* = 0.001). Several plausible reasons may explain this fact. Firstly, nursing practice is an ongoing, patient-related process [32]. Moreover, since nurses care for multiple patients and keep up-to-date records on each patient, their engagement with the EMR system is not limited to specific blocks of activities [33]. In contrast, the work of physicians with EMR systems is usually limited to patient interactions and decision-making activities. The difference in workflow generates an accumulated EMR time among nurses [30]. Secondly, in this study setting, the EMR system has a wider scope of functional activities among nurses (e.g., medication administration, monitoring of vital signs, pre- and post-operative examinations) that require continuous entry and real-time updates. This increases general EMR interaction among nurses compared to physicians [27]. Thirdly, regulatory and institutional procedures can require nurses to fill detailed and redundant documentation forms to comply with safety, quality, and accreditation standards [34]. Other reasons are associated with staffing patterns, delegation, and role distribution [35].

This role-based difference in EMR use may lead to high fatigue, burnout, and job dissatisfaction among nurses [36], suggesting that medical organizations should reassess documentation procedures to ensure a fair digital workload, as well as introduce role-based EMR optimization and supportive interventions to decrease the number of documents that nursing staff must complete.

The difference in the number of hours spent indicates that digital infrastructures have begun to shift the duties of nurses toward data management. Although physicians utilize system when making episodic medical decisions, the average system utilization of 41.73% of working hours among nurses is indicative of a shift in responsibility towards non-direct patient care and more administrative patient care roles. This implies that the system, instead of enabling care, can be a bottleneck. When the time spent managing documentation in the system exceeds 5 h during a 12 h shift, the ability to carry out clinical interventions at the bedside is compromised. That this digital burden persists at the global level indicates that existing versions of digital systems have failed to account for nursing workflows that devote a larger-than-average amount of time to documentation. The data are indicative of the ostensibly unforgiving technical demands of digital systems becoming more substantial than the clinical functions of these systems, thus resulting in system-induced burnout and the erosion of the provider–patient relationship.

### 4.3. Predictors of Extended EMR Use

Gender was found to be a predictive variable of extended EMR use, with female participants spending more time using EMR systems than males. Similarly, it was earlier reported that female physicians spent significantly more time on EHR-related activities during eight-hour planned clinical shifts than male physicians (5.81 versus 5.23 h) [37], indicating that female clinicians might have different communication expectations or documentation patterns that result in higher EMR use.

Regarding nationality, non-Saudi workers were found to dedicate more time to EMRs than their Saudi counterparts. Non-native clinicians might have lengthy documentation times due to language barriers, differences in previous EMR exposure, and institutional norms [38,39]. These factors are probably quite influential in Saudi Arabia, where the healthcare workforce is comparatively multinational [12]. The findings, therefore, indicate that EMR optimization plans must be tailored to local training modules that support professionals from diverse linguistic and cultural backgrounds, thus maximizing efficiency within the entire workforce.

Clinicians with master’s or doctorate degrees were less likely to spend too much time using EMR compared to those with diplomas or bachelor’s degrees. Higher educational attainment has been frequently linked with increased information management and digital literacy skills [40]. Moreover, those with advanced degrees may work as supervisors or decision makers, which could decrease direct data-entry time. User capability and the understanding regarding health informatics have been shown to have a positive impact on system efficiency, leading to faster documentation and reduced cognitive demands [41].

The number of years of professional experience was an important predictor of extended EMR use. Clinicians with moderate-to-extensive experience spent more time on EMRs than less experienced clinicians. Generally, mid-career clinicians both practice and have administrative duties, which increases their documentation workload. Residents and junior physicians have, on average, been reported to spend more time on EHRs with patients at first, becoming more efficient over time, but senior staff tend to outsource documentation [26]. At a Middle Eastern hospital, mid-career professionals were involved in both care provision and supervision, resulting in an increased cumulative EMR interaction time [42]. This observation points to the need to develop EMR systems that effectively facilitate multitasking and supervisory roles.

Clinicians with 5 to 10 years of experience with EMR spent significantly more time using the system. This trend is possibly an indication that users with moderate experience are the most active daily users of EMRs, while beginners are still learning how to use the platform, and the most seasoned users might have learned shortcuts or outsourced some of their tasks. Similar patterns have been reported through audit–log interactions regarding EMR use, with clinicians with intermediate experience having more interactions with a greater number of EMR modules and alerts, in-basket messages, and order entries than other groups [43,44].

A longer EMR training duration (10+ h) was correlated with a longer EMR utilization time. This might seem paradoxical as training is usually anticipated to improve efficiency and reduce time spent on the system. However, it has been shown that extended training leads to more engagement with the advanced features of the system [45].

The fact that gender, nationality, and work experience were found to be significant predictors of EMR use highlights the roles of sociocultural and work-related factors instead of technological constraints in creating digital workload. The longer time allocated to EMR by female and non-Saudi practitioners could be indicative of extra documentation and communication requirements. The qualitative factors that may augment these pressures might include slow system performance, which causes a heterogeneous workforce to work under less-than-ideal conditions. A unified, universal interface, in this case, can be counterproductive when it does not consider various work styles and infrastructure issues, such as low internet connections and computer access.

The negative correlation between the time spent using EMRs and the level of education suggests that more advanced academic preparation can offer cognitive and technical frameworks that allow users to combine information with less effort, despite possible limitations of the system.

The training paradox is that receiving ten or more hours of instruction is associated with a higher level of EMR use, further highlighting the pressures on workflow. Rather than making work easier, broader training can add advanced modules that increase digital work and administrative duties. Thus, training is less of a time-saving mechanism and more of an introduction to more multifaceted documentation mechanisms, which supports the notion that the EMR use is time-intensive.

**Figure 4:**
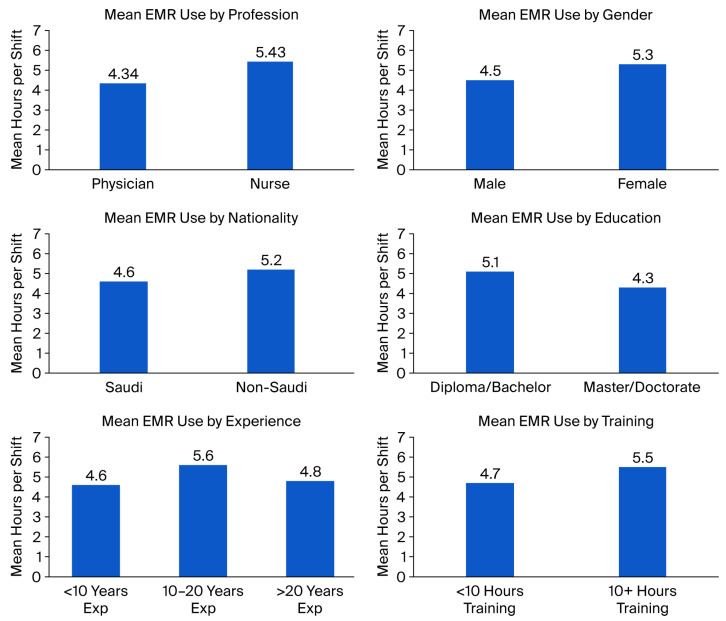
Mean EMR usage over several demographic profiles.

Lastly, the highest EMR usage was recorded among middle-career professionals with between five and ten years of experience, who are expected to connect direct clinical care and supervisory documentation functions. To enhance efficiency, it will probably be necessary to carry out the outgrowth of general training into specific system enhancements, such as system interface simplification, system stability enhancement, and the optimization of database performance, to minimize the time load (Figure 4).

### 4.4. Challenges and Barriers Related to EMR

Infrastructure issues (such as a lack of computers, outdated hardware, and slow internet connectivity) emerged as key barriers to timely and proper EMR use among nurses and physicians. Similar constraints have been reported in a study conducted in Addis Ababa [46] and are associated with documentation delays [47,48] and risks to patient safety and data integrity [49]. These issues impact workflow, decrease productivity, and affect the quality of care, as decisions may be based on outdated or incomplete data [50,51]. This finding highlights the need for investment in hardware enhancement, increasing the number of workstations provided per clinician, and reliable internet services. Additionally, the routine upkeep and upgrading of computers, including maintaining current processing capacity and adequate memory, can reduce latency and downtime. Institutional leadership and IT governance should consider infrastructure support to be a part of EMR usability and data quality. Unless these underlying infrastructure problems are addressed, other interventions, including training, workflow optimization, or user interface improvements, are likely to be limited in their ability to reduce the EMR time burden or improve documentation quality.

System performance and technical issues (such as a slow system, system downtime, and instability) were another obstacle found to affect workflow and hamper the quality of clinical documentation. A previous study conducted in Saudi Arabia indicated that slow system performance was a major cause of user dissatisfaction with EMRs due to its interference with time-sensitive work and distraction from clinical decision-making [52]. In South Africa, regular server outages were shown to be a significant impediment to EMR reliability, forcing clinicians to defer data entry or, at times, resort to paper-based documentation, thereby disrupting the digital record-keeping process [53]. Unexpected or frequent downtimes not only influence productivity but can also threaten patient safety [54], since they interfere with the user workflow and cognitive flow as clinicians must alternate between troubleshooting and patient care activities, which can reduce their efficiency and cause documentation mistakes [55]. System access restrictions were another concern. While restrictions help to secure data, over-enforcement may hinder cooperation and decelerate clinical practices. According to Dutta and Hwang (2020), Saudi healthcare facilities that had restrictive EMR access policies impeded interdepartmental communication and delayed patient-related decision-making, highlighting the need to balance security with usability [56]. The study participants also reported challenges in using the complicated interfaces of EMR systems. Weak user interface design has been directly linked to longer data entry times and perceived workloads in clinicians [57]. Furthermore, frequent system database updates were reported to cause short-term interruptions and forced users to re-learn existing workflows, which sometimes created confusion and opposition, especially when announced without prior notice or necessary training. Similarly, in India, unplanned system upgrades were linked to reduced short-term productivity and higher user frustration [58]. Addressing these issues involves strong technical infrastructure, forward-looking system governance, user-friendly interfaces, and open communication regarding system updates.

A third theme that stood out in the interviews was the perceived lack of support from the IT department in cases where technical problems such as inability to log in, frozen screens or inability to retrieve data occurred. A lack of responsiveness by IT departments has previously been reported to inhibit EMR use and increase frustration among Saudi Arabian clinicians [59]. Similarly, another study stressed that a lack of on-site IT support leads to burnout in users, under-use of the system, and diminished perceived value of EMR systems, particularly when the users have to guess the solution to a problem on their own [40]. In view of this finding, it is urgent to build an effective, well-staffed, and responsive IT support system so that the clinical workflow can run smoothly, documentation delays can be reduced, and users remain confident in EMR technology.

The last theme that emerged related to EMR challenges and barriers was the workflow and professional burdens posed by EMR documentation. Time-consuming documentation was widely reported by participants to be a key contributing factor to work strain. Several clinicians opined that EMR entry procedures were lengthy, comprising several screens and unnecessary fields, taking time away from patient care. This is consistent with a U.S. study that reported that primary-care physicians spent almost half of their working time on EMR-related activities, resulting in exhaustion and reduced patient interaction [44]. Moreover, the interviewees reported increased interaction related to the extra coordination demanded by the EMR systems, such as periodic alerts, communication, and follow-ups. This is consistent with a study that reported a significant portion of screen time of physicians being spent on electronic communication processes, leading to the fragmentation of the workflow and a feeling of constant interruption [36].

Although self-reported data tend to vary with objective time records, the perceived burden of EMR documentation is a very important indicator of system impact. The sub-objectivity of such reports is crucial since perceived workload tends to be a better predictor of professional burnout than the actual time spent using the system. Cognitive fatigue and the feeling that one is technically overburdened is the ultimate cause of clinician dissatisfaction and departure, even if the real temporal commitment is lower than estimated. Thus, the conclusions drawn on this research are not only a chronological indicator of work but also a psychological phenomenon that affects the sustainability of the healthcare workforce in the long term.

Regarding the impact of EMRs, one of the main benefits cited in terms of patient safety was the elimination of handwriting errors. This aligns with earlier studies from Saudi Arabia, which reported that EMRs eliminated illegible handwriting and reduced documentation errors [59,60]. Conversely, participants stated that EMRs have a negative impact on work routine by reducing assessment time and patient bedside time. Similarly, previous research has indicated that EHR systems have impeded nurse–patient communication by restricting opportunities for empathetic interaction [33].

The data on infrastructural and system functioning shows that there is a considerable disconnect between the digital setting and the physical reality. Although electronic records are supposed to achieve efficiency, malfunctioning hardware and unreliable connectivity make technology one of the major barriers in care. It means that technical instability causes not only delays in the documentation process but also the deterioration of the cognitive processes of clinicians who have to cope with troubleshooting and patient needs. Moreover, the conflict between information privacy and organizational access implies that existing institutional policies might focus on the safety of administration rather than clinical responsiveness. These findings emphasize the fact that in the absence of a steady and ready technical base, innovative digital functions are undermined by problems caused by hardware maladjustment and limiting interface structures.

The perceived unsupportiveness of technology and the ensuing excessive documentation are signs that digital tools have not been integrated into the professional social skillset. Regarding the isolation of the clinicians during technical breakdowns, the perceived value of technology and the experience of burnout in the profession increases. Although the system successfully eliminates handwriting errors, a significant loss of bedside interaction and empathy are compensated. This is an indication that the digital model we have today values data integrity more than the human aspect of medicine. The disruptiveness of the workflow due to continuous alerts and redundant entry fields highlights the need for systems that facilitate the clinical process instead of monitoring it. Finally, digital transformation has led to the development of a clinical workload that undermines patient engagement.

The combination of quantitative predictors and qualitative themes creates a complex landscape for the digital documentation load. In particular, the statistical significance of nursing status as a predictor of extended usage is in agreement with qualitative accounts of the redundancy of some administrative duties and the need for continuous real-time surveillance. The paradox of receiving 10 or more hours of training forecasting greater number system interactions is further confirmed by qualitative feedback regarding complex interface designs and the implementation of high-end reporting modules at the expense of time-effectiveness. Moreover, the differences in usage time within the regions are placed into perspective in qualitative reports on irregular technical infrastructure and the different degrees of IT responsiveness in the healthcare environment. These intersecting data points indicate that the temporal burden of EMR is not only a repercussion of the individual qualities of users but also a consequence of the direct relationship between technical system performance and institutional documentation requirements.

### 4.5. Synthesis of Patterns Within the Saudi Context

The Saudi healthcare environment has specific trends that differ from international tendencies, namely workforce diversification and digital maturity. One of the major local features is the strong influence of nationality on system engagement, which highlights the one-of-a-kind multinational structure of Saudi clinical environment. Although the focus of global studies is usually on usability in general, these findings indicate that linguistic and cultural backgrounds are a direct determinant of the length of time spent managing documentation in a region-specific manner. Moreover, the training paradox found in this study signals a distinctive stage in the development of digital infrastructure at the regional level. In this case, contrary to systems established in other countries, more training seems to increase the administrative scope of professionals instead of enhancing time efficiency. The geographical differences between Makkah and Riyadh also indicate that institutional maturity and regional support systems are stronger than individual user characteristics. The results prove that the Saudi situation demands a specific digital approach catering to the professional diversity of the Middle East healthcare industry and the institutional developments unique to it.

The results regarding professional barriers and clinical benefits are conceptually aligned with themes discussed in TAM. Nevertheless, perceived usefulness and perceived ease of use were not assessed or treated as measurable constructs, and the findings should be interpreted as contextual observations rather than indicators of TAM variables. For example, clinicians continued use of EMRs despite reported technical challenges can be interpreted, from a TAM perspective, as consistent with perceived usefulness, particularly when clinicians believe that digital systems support care quality. Conversely, the prevalence of infrastructure constraints and documentation burdens reported by nurses reflects challenges conceptually aligned with issues commonly associated with perceived ease of use, rather than constituting a direct assessment of that construct. These findings suggest that acceptance in Saudi clinical settings is shaped primarily by job-related functional demands and organizational conditions, rather than by individual user preference alone. Viewed within the proposed conceptual framework, this underscores the importance of institutional support and training in bridging the gap between system availability and professional acceptance.

The implications of this study must be explained in the context of the particular institutional context of the Security Forces Hospitals in Riyadh, Dammam, and Makkah. These hospitals, being specialized public healthcare facilities, may have different resources, staffing ratios, and infrastructure standards than those of general public or private hospitals in Saudi Arabia. For example, the gaps in hardware supply and the time delays in IT support could be an institutional policy and not necessarily a common standard among all Saudi healthcare sectors. Therefore, although the digital problems pinpointed are serious, the degree of professional load might be different in other hospital systems managed through other administrative structures.

### 4.6. Research and Practical Implications

The results of this research have profound theoretical implications for the scholarly community and practical implications for health care administrators. In terms of research, these findings expose the need to move beyond generalized evaluations of EMR systems to role-based evaluations. This study indicates that, in the future, other sociotechnical models should be created that consider the specific temporal and technical orientations of nurses and physicians to the digital environment. Moreover, the increasing technological dependence and decline in manual skills upon system breakdowns encourage the growth of a new research field as far as clinical resiliency is concerned. Researchers are also encouraged to further explore the effects of deep-rooted issues with getting used to the impact of the digital workflow on the capacity of a workforce to sustain safety levels during times of unforeseen technical instability.

In practice, these findings can be viewed as a direct guide on how hospital management can enhance clinical efficiency and staff morale. The evident association between infrastructure shortages and professional frustrations implies that institutional investments should focus on the material accessibility and functionality of hardware, as well as on software upgrades. To counter the issue of time burden, companies must cease generic orientation programs and instead provide department-specific training, focusing on workflow streamlining and shortcut exploitation. Moreover, the mentioned loss of bedside interaction requires conducting a critical analysis of documentation requirements to eradicate redundancy in data entry. By minimizing administrative requirements and adding responsiveness to their systems, healthcare facilities can safeguard the provider–patient relationship, so that digital transformation reinforces anew the humanistic essence of clinical care.

### 4.7. Study Limitations, Strengths, and Future Research

A key strength of this study is its mixed-methods design, which enabled us to gain a better insight into the phenomenon. This research was carried out in three large Security Forces Hospitals in Riyadh, Dammam and Makkah, which enhanced the overall transferability of the findings and the possibility of comparison between various healthcare staff working in the same organizational context and EMR [61]. This study’s large and diverse target population of nurses and physicians allowed for comparison between professional groups and highlighted role-based disparities in EMR utilization. Using a multistage sampling method increased the representativeness of the sample [62]. The sample size was also sufficiently large to perform rigorous analytical processes, and validated instruments were used, which further enhanced the strength of the quantitative findings.

This study has several limitations that should be considered.

➢ First, this study involved several sites; however, all of them were Security Forces Hospitals. This restriction limits extrapolation to other healthcare organizations in Saudi Arabia, especially non-military or private hospitals with different EMR systems, administrative organizations, or resources.
➢ Second, the quantitative data were self-reported, which may introduce recall bias or social desirability bias despite anonymity measures [63].
➢ Third, the cross-sectional design does not allow for determining causality [64]. The qualitative element relied on short interviews with a small sample of participants, which prevented the attainment of more in-depth insights into the psychological, cultural, or organizational processes that affect EMR use [65]. Inconsistencies in EMR usage measurement across departments, patient loads, and staff roles may have also affected self-reported time, as no objective system-recorded data were used [66].
➢ Finally, a lack of time and available resources restricted the extent of data collection and data analysis, preventing the examination of other variables such as job satisfaction, burnout, or patient outcomes.
➢ Another major limitation of this research is the reliance on self-reported data to measure EMR usage duration, which may introduce recall and perception bias. Participants might overestimate their time spent on digital tasks due to the high cognitive load and professional frustration associated with documentation, leading to an inflation of the perceived administrative burden. While self-reporting is a common and practical approach in largescale clinical studies, it remains a subjective measure compared to objective audit log data. To mitigate this limitation, future research should integrate system-generated timestamps with survey data to provide a more precise calculation of active engagement time. Despite this potential for bias, the current findings offer valuable insights into professional experience of EMR burden and reflect the subjective reality of the healthcare workforce, which is a critical component of professional satisfaction and system acceptance.
➢ In addition, the relatively small sample size for the qualitative phase is a concerning limitation. Although the research team determined that data saturation had been reached as no new themes emerged during the final interviews, the results may not fully capture the diverse perspectives of the entire healthcare workforce in Saudi Arabia. Future research involving a larger and more varied group of participants across different regions could further validate these findings and offer additional insights into EMR-related challenges.
➢ Moreover, this research’s focus on a specific public hospital system may limit the generalizability of the results to the broader Saudi national context. The resource levels and technical support structures at Security Forces Hospitals might differ from those in the private sector or smaller community clinics. Therefore, the findings should be viewed as a representative case study of a major public health system rather than a comprehensive national survey.

Future research should include longitudinal evaluations of EMR use to examine the effects of system upgrades or policy interventions on time burden over time. Moreover, comparative analysis of public and private hospitals in Saudi Arabia may identify contextual and organizational factors that influence EMR efficiency. Qualitative studies that explore the emotional and cognitive effects of EMR-related workload can enhance our understanding of burnout and job satisfaction among healthcare professionals.

## 5. Conclusions

This study highlights significant variation in EMR use among healthcare professionals, with nurses bearing a disproportionately high documentation burden. Sociodemographic and professional characteristics shape EMR engagement, underscoring the inherently sociotechnical nature of digital work. Despite recognizing the value of EMRs for patient safety and clinical practice, participants identified persistent infrastructure gaps, system instability, limited IT support, and workflow disruption as major barriers. These findings point to clear institutional priorities: invest in reliable digital infrastructure, expand timely and responsive IT support, allocate protected documentation time, and provide continuous, role specific EMR training to reduce administrative burden and better support frontline staff. At the policy level, the results reinforce the need for national standards that promote interoperability, mandate usability requirements, strengthen data governance, and ensure structured, competency based EMR training. Future research should explore longitudinal patterns of EMR adoption, compare outcomes across diverse care settings, and evaluate emerging technologies, such as AI-enabled documentation and mobile interfaces, for their potential to reduce workload and enhance clinician–patient interaction.

## Figures and Tables

**Table 1:** The demographic characteristics of nurses and physicians (*n* = 503).

	Total Participants (*n* = 503)		Physicians (*n* = 162)		Nurses (*n* = 341)	
	Number	%	Number	%	Number	%
Sex						
Male	165	32.8	93	57.4	72	21.1
Female	338	67.2	69	42.6	269	78.9
Age (years)						
<30	82	16.3	40	24.7	42	12.3
30–39	226	44.9	59	36.4	167	49
40–49	122	24.3	48	29.6	74	21.7
≥50	73	14.5	15	9.3	58	17
Nationality						
Saudi	191	38	117	72.2	74	21.7
Non-Saudi	312	62	45	27.8	267	78.3
Position of Physicians						
Intern	4	0.8	4	2.5	-	-
Resident	69	13.7	69	42.6	-	-
Fellow	10	2	10	6.2	-	-
Specialist	36	7.2	36	22.2	-	-
Consultant	38	7.6	38	23.5	-	-
General Practitioner	5	1	5	3.1	-	-
Primary Specialty for Physicians						
Anesthesia	7	1.4	7	4.3	-	-
Cardiology	4	0.8	4	2.5	-	-
Emergency medicine	5	1	5	3.1	-	-
Family medicine	21	4.2	21	13	-	-
General practitioner	5	1	5	3.1	-	-
Intensivist	4	0.8	4	2.5	-	-
Internal medicine	20	4	20	12.3	-	-
Neurosurgery	8	1.6	8	4.9	-	-
OB/Gynecology	13	2.6	13	8	-	-
Ophthalmology	3	0.6	3	1.9	-	-
Orthopedic	11	2.2	11	6.8	-	-
Pediatric	44	8.7	44	27.2	-	-
Surgery	17	3.4	17	10.5	-	-
Position of Nurses						
Clinical nurse specialist	82	16.3	-	-	82	24
Frontline	209	41.6	-	-	209	61.3
(Direct patient care provider)						
Nurse manager/supervisor	50	9.9	-	-	50	14.7
Work Setting for Nurses						
Hospital (inpatient)	159	31.6	-	-	159	46.6
Hospital (outpatient)	43	8.5	-	-	43	12.6
Emergency room (ER)	35	7	-	-	35	10.3
Intensive care unit (ICU)	98	19.5	-	-	98	28.7
Transitional care unit (TCU)	1	0.2	-	-	1	0.3
Surgical/operating room	29	5.8	-	-	29	8.5
Primary healthcare center	14	2.8	-	-	14	4.1
Nursing admin	9	1.8	-	-	9	2.6
Burn unit	1	0.2	-	-	1	0.3
Neonatal intensive care unit (NICU)	1	0.2	-	-	1	0.3
Burn care unit (BCU)	1	0.2	-	-	1	0.3
Highest Level of Education Certificate in Profession						
Diploma	46	9.1	3	1.9	43	12.6
Bachelor’s degree	327	65	73	45.1	254	74.5
Master’s degree	83	16.5	43	26.5	40	11.7
Doctorate (PhD or Equivalent)	47	9.3	43	26.5	4	1.2
Years of Experience in Healthcare						
<1 year	16	3.2	5	3.1	11	3.2
1–4 years	86	17.1	53	32.7	33	9.7
5–9 years	109	21.7	34	21	75	22
10–19 years	181	36	35	21.6	146	42.8
≥20 years	111	22.1	35	21.6	76	22.3
Years of Experience in Healthcare Setting Utilizing EMR Systems						
<1 year	30	6	11	6.8	19	5.6
1–4 years	200	39.8	80	49.4	120	35.2
5–9 years	142	28.2	34	21	108	31.7
10–19 years	107	21.3	32	19.8	75	22
≥20 years	24	4.8	5	3.1	19	5.6
Region of Work						
Riyadh	252	50.1	101	62.3	151	44.3
Makkah	132	26.2	31	19.1	101	29.6
Dammam	119	23.7	30	18.5	89	26.1

**Table 2:** The average number of hours of EMR use among physicians and nurses (*n* = 503).

Question	Total Participant		Physicians		Nurses	
	Number	%	Number	%	Number	%
On average, how many hours per shift/day do you spend using the EMR system?						
Less than 1 h	17	3.4	4	2.5	13	3.8
1–2 h	49	9.7	23	14.2	26	7.6
3–4 h	125	24.9	61	37.7	64	18.8
5–6 h	98	19.5	43	26.5	55	16.1
More than 6 h	214	42.5	31	19.1	183	53.7
What specific tasks do you use the EMR system for?						
Reviewing test results	372	74	142	87.7	230	67.4
Documenting patient histories	348	69.2	140	86.4	208	61
Patient admission	319	63.4	105	64.8	214	62.8
Updating progress notes	305	60.6	144	88.9	161	47.2
Nursing notes	305	60.6	7	4.3	298	87.4
Nursing initial assessment	288	57.3	8	4.9	280	82.1
Discharge process	285	56.7	100	61.7	185	54.3
Writing reports	272	54.1	114	70.4	158	46.3
Updating treatment plans	244	48.5	130	80.2	114	33.4
Communication with other healthcare providers “Request/consultation”	231	45.9	118	72.8	113	33.1
Nursing care plan	228	45.3	4	2.5	224	65.7
Entering diagnostic data	203	40.4	120	74.1	83	24.3
Prescribing medications	197	39.2	144	88.9	53	15.5
Other	19	3.8	3	1.9	16	4.7
Have you received formal training in using the EMR system?						
No	65	12.9	34	21	31	9.1
Yes	438	87.1	128	79	310	90.9
How many hours of EMR training have you received?						
Zero	65	12.9	30	18.5	35	10.3
Less than 5 h	246	48.9	88	54.3	158	46.3
5–10 h	126	25	33	20.4	93	27.3
More than 10 h	66	13.1	11	6.8	55	16.1
What are the primary challenges you face when using the EMR system?						
Lack of adequate training	141	28	73	45.1	68	19.9
Slow system performance	239	47.5	64	39.5	175	51.3
System crashes or errors	169	33.6	66	40.7	103	30.2
Difficulty navigating the system	122	24.3	61	37.7	61	17.9
Time-consuming data entry	258	51.3	110	67.9	148	43.4
Lack of user-friendly interface	123	24.5	51	31.5	72	21.1
Disrupts workflow	143	28.4	54	33.3	89	26.1
Difficulty in communication with other healthcare providers via the EMR	110	21.9	44	27.2	66	19.4
Lack of adequate technical support	157	31.2	54	33.3	103	30.2
Other	16	3.2	5	3.1	11	3.2

**Table 3:** Total hours of EMR use per day among physicians and nurses.

		All		Nurse		Physician	
Time Category (Hour)	Approx. Midpoint (Hours/Day)	Number	Total (Hours/Day)	Number	Total (Hours/Day)	Number	Total (Hours/Day)
<1	0.5	17	8.5	13	6.5	4	2
1–2	1.5	49	73.5	26	39	23	34.5
3–4	3.5	125	437.5	64	224	61	213.5
5–6	5.5	98	539	55	302	43	236.5
>6	7	214	1498	183	1281	31	217

**Table 4:** Mean monthly hours of EMR use for physicians and nurses and its percentages out of total monthly working hours.

Position	Work Hours per Day	Work Days per Week	Total Work Hours per Month	Mean of EMR Usage Hours per Day	Total of EMR Usage Hours per Month	% of EMR Usage per Month out of the Total Monthly Working Hours	*p*-Value
Nurses	12	4	208	5.43 ± 2.03	86.80	41.73	0.001 *
Physicians	12	4	208	4.34 ± 1.87	69.44	33.38	

* Significant *p* value; *p* value was calculated for the mean of EMR usage per day between nurses and physician.

**Table 5:** Predictors of extended hours of use in relation to demographic characteristics using ordinal logistic regression.

		Odds Ratio	95% CI		*p*-Value
			Lower	Upper	
Gender	Male	Reference Group			
	Female	3.08	2.17	4.36	0.001 *
Age	Below 30	Reference Group			
	30–40	1.94	0.89	2.78	0.005 *
	40–50	1.81	1.09	3.04	0.022 *
	50 above	1.57	1.23	3.07	0.118
Nationality	Saudi	Reference Group			
	Non-Saudi	2.92	0.74	1.41	0.001 *
Position	Physician	Reference Group			
	Nurse	2.98	2.12	4.20	0.001 *
Education	Diploma	Reference Group			
	Bachelor	0.69	0.39	1.27	0.241
	Master	0.50	0.25	0.99	0.047 *
	Doctorate	0.28	0.14	0.61	0.001 *
Experience	<1 year	Reference Group			
	1–5 year	2.66	0.99	7.19	0.053
	5–10 year	5.05	1.88	13.55	0.001 *
	10–20 year	5.23	1.99	13.76	0.001 *
	20+ year	4.15	1.55	11.15	0.005 *
EMR Experience	<1 year	Reference Group			
	1–5 year	1.32	0.66	2.69	0.429
	5–10 year	2.13	1.03	4.41	0.041 *
	10–20 year	2.08	0.99	4.41	0.055
	20+ year	2.30	0.85	6.26	0.101
EMR Training	0	Reference Group			
	<5 h	1.00	0.62	1.65	0.978
	5–10 h	1.55	0.92	2.76	0.095
	10+ h	2.33	1.22	4.47	0.010 *
Region	Riyadh	Reference Group			
	Makkah	1.54	1.05	2.26	0.026 *
	Dammam	1.08	0.72	1.64	0.690

* Significant *p* value.

**Table 6:** Mean score of perceived challenges and barriers facing physicians and nurses when using the EMR system.

Question		Likert Scale Feedback					Mean, Standard Deviation and *p* Value			
		Strongly Disagree *n* (%)	Disagree *n* (%)	Neutral *n* (%)	Agree *n* (%)	Strongly Agree *n* (%)	All Mean ± SD	Physicians Mean ± SD	Nurses Mean ± SD	*p* Value
1.	I received adequate training in using EMR	53 (10.5)	54 (10.7)	146 (29)	174 (34.6)	76 (15.1)	3.33 ± 1.17	3.04 ± 1.21	3.47 ± 1.13	<0.001 *
2.	I always receive immediate support when facing technical issues with the EMR system	49 (9.7)	72 (14.3)	175 (34.8)	152 (30.2)	55 (10.9)	3.18 ± 1.11	3.01 ± 1.15	3.26 ± 1.09	0.018 *
3.	In my opinion, EMR documentation has significantly increased the quality of patient care	35 (7.0)	19 (3.8)	137 (27.2)	185 (36.8)	127(25.2)	3.70 ± 1.1	3.91 ± 1.01	3.6 ± 1.13	0.003 *
4.	EMR documentation affects my ability to interact with patients directly	42 (8.3)	108 (21.5)	157 (31.2)	137 (27.2)	59 (11.7)	2.87 ± 1.13	2.77 ± 1.23	2.92 ± 1.08	0.158
5.	Performing tasks on the EMR takes more time compared to direct patient care	42 (8.3)	85 (16.9)	159 (31.6)	131 (26.0)	86 (17.1)	2.73 ± 1.17	2.48 ± 1.22	2.85 ± 1.13	0.001 *
6.	Utilizing the EMR system for performing tasks has enhanced my job satisfaction	32 (6.4)	44 (8.7)	177 (35.2)	181 (36.0)	69 (13.7)	3.42 ± 1.04	3.44 ± 1.05	3.41 ± 1.04	0.780
7.	I believe that my age affects my ability in using the EMR system	156 (31.0)	152 (30.2)	112 (22.3)	62 (12.3)	21 (4.2)	3.72 ± 1.15	3.62 ± 1.26	3.76 ± 1.1	0.186
8.	I believe that my years of experience affect my ability in using the EMR system	100 (19.9)	156 (31.0)	103 (20.5)	98 (19.5)	46 (9.1)	3.33 ± 1.25	3.09 ± 1.29	3.44 ± 1.22	0.003 *
9.	My working position (title) influences the time spent on EMR system (e.g., junior, senior, intern)	80 (15.9)	137 (27.2)	125 (24.9)	112 (22.3)	49 (9.7)	3.17 ± 1.22	2.69 ± 1.3	3.4 ± 1.11	0.001 *
10.	My health care setting affects the time spent on EMR system	52 (10.3)	100 (19.9)	169 (33.6)	133 (26.4)	49 (9.7)	2.95 ± 1.13	2.61 ± 1.05	3.11 ± 1.13	0.001 *
11.	The time spent using EMR system has a positive impact on my job satisfaction	36 (7.2)	70 (13.9)	166 (33)	168 (33.4)	63 (12.5)	3.30 ± 1.08	3.3 ± 1.13	3.3 ± 1.06	0.933

* Significant *p* value. Likert scale with 5 points, where 1 = strongly disagree, 2 = disagree, 3 = neutral, 4 = agree, and 5 = strongly agree.

**Table 7:** Multiple linear regression analysis for assessing the predictors of perceived challenges and barriers toward EMR use among healthcare professionals.

Predictor	β	t	*p* Value
Gender	0.008	0.174	0.862
Age	0.054	0.788	0.431
Nationality	0.075	1.259	0.209
Position	0.163	2.877	0.004 *
Education level	0.038	0.744	0.457
Years in healthcare	0.012	0.146	0.884
EMR experience	−0.033	−0.621	0.535
Work region	−0.110	−2.401	0.017 *
Hours of EMR training	0.173	3.826	<0.001 *

R^2^ = 0.118, F = 7.298, *p* < 0.001. * Significant *p* value.

**Table 8:** A summary of EMR barriers and challenges: themes, codes, and sub-codes.

Theme: EMR Barriers and Challenges	
Code	Subtheme
Lack of Computers	Infrastructure Issues
Computer Not Up to Date	
Slow Internet	
Slow System	System Performance and Technical Issues
System Down	
System Stability	
System Access Restriction	
User Interface Issue	
System Database Update	
Lack of support from the IT department	Lack of Support
Time-Consuming	Workflow and Professional Burden
Increase in Interactions	

**Table 9:** A summary of the impacts of EMRs.

Theme: EMR Impact	
Code	Subtheme
More Attached with Patient	Improved Professional Practice
Organized and Structured	
Secured	Improved Patient Safety
Solve Handwriting Issue	
Lost Patient Bedside Time	Work Routine Adjustment
Reduce Assessment Time Allocation	
Difficulty Reverting to Manual Method	

## Data Availability

Data are contained within the article or the Supplementary Materials.

## Supplementary Materials

The following supporting information can be downloaded at https://www.mdpi.com/article/10.3390/healthcare14040441/s1.
