# Supplementary material for: Time Burden of Electronic Medical Records on Nurses and Physicians in Saudi Arabia: Occurrence, Predictors, and Challenges—A Mixed-Methods Study
Source: Healthcare (Basel). 2026 Feb 9;14(4):441. doi: 10.3390/healthcare14040441 (PMC12940217; doi:10.3390/healthcare14040441)
Supplement: Supplementary file 1 [file healthcare-14-00441-s001.zip › healthcare-4067324-supplementary.pdf]

Supplementary Material

1 Electronic Medical Records (EMR) Time Burden Questionnaire

1.1 Demographics

1. **Gender:**
   - o Male
   - o Female
2. **Age:**
   - o Below 30
   - o 30 to Below 40
   - o 40 to Below 50
   - o 50 and Above
3. **Nationality:**
   - o Saudi
   - o Non-Saudi (please specify)
4. **Position: <u>Physician Only</u>**
   - o Not Applicable (I am a Nurse)
   - o Intern
   - o Resident
   - o Fellow
   - o Specialist
   - o Consultant
   - o Other (please specify): ________
5. **What is your primary Specialty – <u>Physicians</u>- (e.g. Orthopedic, GS, Neuro...?)**

-
- Not Applicable (I am a Nurse)
- Other (please specify):____________

6. **Position: <u>Nurses Only</u>**
   - o Not Applicable (I am a physician)
   - o Nurse manager/supervisor
   - o Front line (Direct patient care provider)
   - o Clinical nurse specialist
   -
   - o Other (please specify): ________
7. **Work setting <u>(Nurses)</u>** (*You may select multiple options*)
   - o Not Applicable (I am a physician)

- Hospital (inpatient)
- Hospital (outpatient)
- Emergency Room (ER)
- Intensive Care Unit (ICU)
- Surgical/Operating Room
- Primary Healthcare center
- Other (please specify): ________

8. **Highest level of education certificate in your profession?**
   - Diploma
   - Bachelor's degree
   - Master's degree
   - Doctorate (PhD or equivalent)
   - Others: ________

9. **Years of experience in healthcare:**
   - Less than 1 year
   - 1 to less than 5 years
   - 5 to less than 10 years
   - 10 to less than 20 years
   - 20 years and above
10. **Years of Experience in healthcare setting utilizing EMR?**
   - Less than 1 year
   - 1 to less than 5 years
   - 5 to less than 10 years
   - 10 to less than 20 years
   - 20 years and above
11. **In what region do you work?**
   - Riyadh
   - Makkah
   - Dammam
   - Other (please specify): ________

1.2 EMR Usage

12. **On average, how many hours per Shift/day do you spend using the EMR system?**

- Less than 1 hour
- 1-2 hours
- 3-4 hours
- 5-6 hours
- More than 6 hours

13. **What specific tasks do you use the EMR system for? (You may select multiple options)**

- Patient admission
- Documenting patient histories
- Updating progress notes
- Entering diagnostic data
- Updating treatment plans
- Prescribing medications
- Communication with other healthcare providers “Request/consultation”
- Reviewing test results
- Writing reports
- Discharge process
- Nursing initial assessment
- Nursing care plan
- Nursing notes
- Other (please specify): ________

1.3 EMR Training and Support

14- Have you received formal training in using the EMR system?

- o Yes
- o No

15- How many hours of EMR training have you received?

- None
- Less than 5 hours
- 5-10 hours
- More than 10 hours

16. What are the primary challenges you face when using the EMR system? (Select all that apply)

- Lack of adequate training
- Slow system performance
- System crashes or errors
- Difficulty navigating the system
- Time-consuming data entry
- Lack of user-friendly interface
- Disrupts workflow
- Difficulty in communication with other healthcare providers via the EMR
- Lack of adequate technical support
- Other (please specify): ________

1.4 Perceptions of EMR Use:

To answer the following questions (17-27) please use the likert scale from 1-5; where is 1 strongly disagree, 2 disagree ,3 neutral ,4 agree and 5 strongly agree)

17. I received adequate training in using EMR?

☐ 1 ☐ 2 ☐ 3 ☐ 4 ☐ 5

18. I always receive immediate support when facing technical issues with the EMR system?

☐ 1 ☐ 2 ☐ 3 ☐ 4 ☐ 5

19. In my opinion, EMR documentation has significantly increased the quality of patient care?

☐ 1 ☐ 2 ☐ 3 ☐ 4 ☐ 5

20. EMR documentation affects my ability to interact with patients directly?

☐ 1 ☐ 2 ☐ 3 ☐ 4 ☐ 5

21. Performing tasks on the EMR takes more time compared to direct patient care.

☐ 1 ☐ 2 ☐ 3 ☐ 4 ☐ 5

22. Utilizing the EMR system for performing tasks has enhanced my job satisfaction

☐ 1 ☐ 2 ☐ 3 ☐ 4 ☐ 5

23- I believe that my age affects my ability in using the EMR system?

☐ 1 ☐ 2 ☐ 3 ☐ 4 ☐ 5

24- I believe that my years of experience effect my ability in using the EMR system .

☐ 1 ☐ 2 ☐ 3 ☐ 4 ☐ 5

25- My working position (title) influence the time spent on EMR system (e.g., junior, senior, intern) .

☐ 1 ☐ 2 ☐ 3 ☐ 4 ☐ 5

26. **My health care setting affects the time spend on EMR system.**

☐ 1 ☐ 2 ☐ 3 ☐ 4 ☐ 5

27- The time spent using EMR system has a positive impact on my job satisfaction.

☐ 1 ☐ 2 ☐ 3 ☐ 4 ☐ 5

1.5 Suggestions and Recommendations

28-Are there any missing information or incomplete training that can be added during training on using the EMR system?
(Open-ended question)

29-From your perspective, how can the difficulties associated with EMR usage be resolved in your hospital or healthcare setting?
(Open-ended question)

30-Any other comments or suggestions regarding EMR use in your healthcare setting?
(Open-ended question)
